# Multivariable flexible modelling for estimating complete, smoothed life tables for sub-national populations

**DOI:** 10.1186/s12889-015-2534-3

**Published:** 2015-12-16

**Authors:** Bernard Rachet, Camille Maringe, Laura M. Woods, Libby Ellis, Devon Spika, Claudia Allemani

**Affiliations:** Cancer Survival Group, Department of Non-Communicable Disease Epidemiology, London School of Hygiene and Tropical Medicine, Keppel street, London, WC1E 7HT UK

**Keywords:** Life tables, Model life tables, Mortality rates, Life expectancy, Generalised linear model, Cubic splines, Deprivation, Small areas

## Abstract

**Background:**

The methods currently available to estimate age- and sex-specific mortality rates for sub-populations are subject to a number of important limitations. We propose two alternative multivariable approaches: a relational model and a Poisson model both using restricted cubic splines.

**Methods:**

We evaluated a flexible Poisson and flexible relational model against the Elandt-Johnson approach in a simulation study using 100 random samples of population and death counts, with different sampling proportions and data arrangements. Estimated rates were compared to the original mortality rates using goodness-of-fit measures and life expectancy. We further investigated an approach for determining optimal knot locations in the Poisson model.

**Results:**

The flexible Poisson model outperformed the flexible relational and Elandt-Johnson methods with the smallest sample of data (1%). With the largest sample of data (20%), the flexible Poisson and flexible relational models performed comparably, though the flexible Poisson model displayed a slight advantage. Both approaches tended to underestimate infant mortality and thereby overestimate life expectancy at birth. The flexible Poisson model performed much better at young ages when knots were fixed *a priori*. For ages 30 and above, results were similar to the model with no fixed knots.

**Conclusions:**

The flexible Poisson model is recommended because it derives robust and unbiased estimates for sub-populations without making strong assumptions about age-specific mortality profiles. Fixing knots* a priori *in the final model greatly improves fit at the young ages.

## Background

Life tables are sets of age-specific mortality rates for a given population or sub-population. They are an important demographic tool for social sciences as well as many other fields of scientific research, and are the basis for the calculation of life expectancy. Complete life tables, which contain estimates of the probabilities and rates of death by single year of age and sex, also provide crucial information for the estimation of net survival and crude probability of death from a given disease of interest.

Complete life tables are published by sex at a national level on a regular basis. However, such life tables are incomplete or unavailable for many middle and low-income countries. Furthermore, because background mortality can vary widely, either geographically, by socio-economic level, or by ethnicity, it is desirable to be able to produce life tables specific to these populations in order to better understand how mortality varies within them [1].

Since life tables for sub-populations are, by definition, based upon smaller populations, observed mortality rates tend to be more unstable. Moreover, both the original data (deaths and population) and consequently the life tables derived from them are commonly abridged, i.e. defined for age groups (0, 1–4, 5–9 … 80–84, 85+) rather than for every single year of age. This is frequently due to confidentiality restrictions on obtaining data for minority groups or for small geographical areas.

Model life table methods were originally formulated to estimate sets of mortality rates for the entire life course (i.e. ages 0–85, 99 or above) in settings where demographic data were unavailable or unreliable [2]. They can also be used to estimate complete (i.e. by single year of age) sets of mortality rates from abridged data. To achieve this end, several approaches have since been applied, including relational [2, 3] and interpolation techniques [4, 5], or the application of one of these standard methods to data on the same population obtained from different data sources [6], in order to produce reliable, smoothed sets of mortality rates for small populations.

There are three distinct limitations common to all of these approaches. First, for all these approaches, estimates of mortality for the population of interest are themselves derived from the proportion left alive at each age. None of these approaches uses actual counts of populations and deaths, which means that the variance of the mortality rates cannot be estimated. Second each of these methods relies heavily upon external information about the pattern of mortality by age, either through the requirement for a reliable standard population life table, or through pre-specified coefficients which are used to generate the age- and sex-specific mortality profile. Although this can be deemed necessary in settings where data quality is known to be poor, it can be limiting in settings where the data is of reliable quality. Third, each of these approaches is univariable, which can lead to unstable estimates when applied to small populations. In our experience, these constraints lead to estimated mortality rates which do not closely fit the observed mortality rates in small sub-populations or in national populations with more unusual mortality profiles [7].

We here propose and evaluate two alternative approaches to the derivation of complete life tables for sub-populations. Both enable the joint modelling of several covariables and are more flexible than existing methods. One also avoids strong *a priori* assumptions about the underlying shape of the population’s mortality profile (the distribution of the deaths by age), and allows to take fully into account the variance, since estimates are modelled directly on observed counts of deaths and populations. In section 2 we describe existing approaches and present our two proposed alternative models. The third section describes the simulation study we used to evaluate these two methods against the best existing approach. The fourth section details an attempt to address the issue of knot placement, encountered during the simulation study. Finally we discuss the advantages and disadvantages of the various approaches, and make some recommendations for further methodological work.

## Methods

Our aim was to develop a more reliable and flexible method to construct sub-population life tables than the best methods currently available. We define sub-population life tables as life tables specific to a variable other than age and sex which is available at population level, for example, geography, ethnicity or socio-economic status.

### Existing approaches

Bailli *et al.* [8] have previously evaluated the univariable approaches to the problem of producing smooth, complete life tables from abridged data and for small populations. They concluded that the Elandt-Johnson interpolation method [4] was either equivalent or superior in terms of goodness-of-fit in comparison to Kostaki [9], Brass logit [2] and the Akima spline [5] methodologies.

The Elandt-Johnson method derives the number of survivors from the abridged life table using different interpolation functions according to age. Linear interpolation is applied only up to 75 years of age, while the Gompertz survival distribution [10] is used for older ages. The linear interpolation ensures that the abridged raw values are kept for the corresponding ages in the complete life tables. The Elandt-Johnson method can only be applied to abridged (grouped age) data.

### Alternative approaches

#### Flexible relational model

We used Ewbank’s well-established model life table technique [3] as the basis of a more flexible model life table approach. We performed a linear regression of the *logit* transformation of the observed survivorship function (*l*_*x*_) values over a similarly transformed survivorship function of a reliable standard population $\left({l}_{x_s}\right)$. Co-variables such as deprivation can be included in this model. We evaluated interactions between deprivation and age using restricted cubic regression splines. The advantage of this approach over the traditional Ewbank four parameter model is that it is multivariable and also more flexible. However, it still relies on an external standard for values of ${l}_{x_s}$ and is not based upon the raw deaths and population data. It is possible to apply this model to both abridged and complete data.

$$ logit\left({l}_{x,i}\right)=f\left( logit\left({l}_{x_s}\right)\right)+{\displaystyle \sum_{i=2}^5}{\beta}_i deprivatio{n}_i+g(agedep) $$

where: *x* denotes age and *i* indexes the deprivation category, *l*_*x*,*i*_ denotes the age- and deprivation-specific survivorship function in the observed population, ${l}_{x_s}$ denotes the age-specific survivorship function in the standard population, *agedep* denotes the interaction between age and deprivation, and *f* and *g* denote restricted cubic spline functions.

#### Flexible Poisson model

We also defined an alternative, novel, approach in which age-specific death counts were modelled within the generalised linear model framework, with a Poisson error, a log link and the person-years at risk as the offset [11]. Age represents the timescale variable. Co-variables can be incorporated in this multivariable model and interactions can be fitted with restricted cubic splines. The advantage of this approach over existing methods is that it is multivariable, very flexible and makes use of observed counts of deaths and populations at all ages from birth to 100 years. Additionally, this approach is unique amongst all those described because it does not make any assumptions about the underlying shape of the mortality rates by age and other co-variables. This model can be applied to both abridged and complete data.

$$ log\left({d}_{x,i}\right)={\beta}_0+f(x)+{\displaystyle \sum_{i=2}^5}{\beta}_i deprivatio{n}_i+g(agedep)+ log\left(pyr{s}_{x,i}\right) $$

where, additionally, *d*_*x*,*i*_ denotes the age- and deprivation-specific counts of death in the population and *pyrs*_*x*,*i*_ denotes the age- and deprivation-specific number of person-years at risk in the population.

## Results

### Simulation study

The simulation study was designed to evaluate whether the flexible relational and Poisson models performed better than the Elandt-Johnson method when producing smooth, complete life tables for sub-national populations.

#### Baseline data

We obtained census-derived population counts for electoral wards in England in 2001 from the Office for National Statistics. We calculated the 2001 Townsend index [12] for each of the 7707 electoral wards that existed in England in 2001. The Townsend index is a measure of deprivation, based upon four household characteristics (unemployment, overcrowding, car ownership, home ownership) collected in the decennial census. The electoral wards were then ranked by the index and divided into five categories according to the quintiles of the wards distribution ranging from least deprived (the 20% of wards with the lowest scores) to most deprived (the 20% of wards with the highest scores).

#### Generation of mortality rates

We have previously published complete and smoothed sex-specific life tables for England as a whole and by deprivation [7], based on the 1991-census data and using the conventional Ewbank model life table approach [3]. In these life tables the effect of deprivation upon the age-specific mortality was non-proportional.

We applied these sets of age-, sex- and deprivation-specific mortality rates to the population counts for England in 2001 in order to obtain the number of deaths by single year of age, sex and deprivation that would have occurred in 2001 had these deprivation-specific mortality rates applied.

We then drew, from these counts of populations and deaths, 100 random samples of 20, 10 and 1% of individuals, corresponding to about 1 million, 500,000 and 50,000 men or women by deprivation category, respectively (Table 1). The corresponding numbers of deaths range from around 12,000 (in 20% random samples) to 600 (1% samples).

**Table 1:** Average^a^ number of people and deaths in each deprivation group

		Reference population	Samples		
			20%	10%	1%
Population (denominator)	Males	4 784 216	956 510	478 270	47 931
	Females	5 042 120	1 008 268	504 385	50 646
Deaths (numerator)	Males	60 840	12 065	6 070	600
	Females	58 444	11 691	5 833	587

^a^Averaged across 100 samples

Population and mortality data are not always available by single year of age and/or up to 100 years of age. To reflect this, we then rearranged the data according to four pre-specified scenarios; (i) abridged (grouped age) data up to 80 (0, 1–4, 5–9, … 75–79, 80+), (ii) abridged (grouped age) data up to 95 (0, 1–4, 5–9, … 90–94, 95+), (iii) counts of deaths and populations by single year of age up to 80 years (with information for the age group 80+), and (iv) counts of deaths and populations by single year of age up to 100 years. The first of these scenarios is that with the least detailed information, but is still the most common scenario across the world [13–16].

**Fig. 1:**
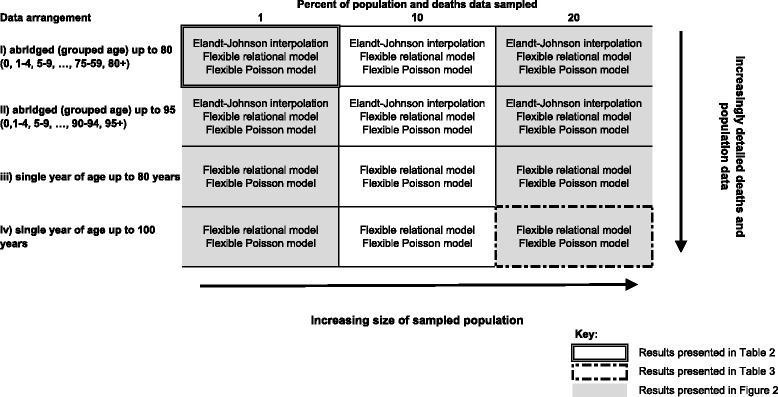
Diagram illustrating the different data samples and arrangements examined in the simulation study, and which of the methods were applied to each

We thus derived 2400 separate data sets containing age specific counts of populations and deaths (100 simulations for men and women x 3 sampling proportions x 4 data rearrangements, Fig. 1). When abridged data were used, the age value of the age group was centred *a priori*.

#### Application of the methods

##### Flexible relational model

We fitted the flexible relational model to the observed *l*_*x*_ values separately for each sex. We included deprivation as a covariable. We used the 1998–2000 national life tables for England as the standard population $\left({l}_{x_s}\right)$ [17]. Separate life tables for each of the five deprivation categories were derived from the model.

##### Flexible Poisson model

We fitted the flexible Poisson model to counts of deaths and populations separately for men and women. As the relationships between the death rates, deprivation and age were likely to be non-proportional, an interaction term between age and deprivation was systematically incorporated in the initial model. All variables initially incorporated in the model, including the interaction, were forced. The selection of the model and the shape of the flexible functions were assessed using the algorithm embedded in the Stata command *mvrs* (see Royston *et al.* [11] for more details), based on the Akaike information criterion (AIC) [18]. Restricted cubic splines are flexible polynomial functions that are joined at locations termed ‘knots’. They are constrained to be linear beyond the locations of the boundary knots. The number of knots was selected *a priori* – 6 for age and 3 for the interaction – while their location was defined by the model according to the percentiles of the death distribution across all ages. Such numbers of knots are a good compromise between having enough flexibility to capture successive inflections of the age-specific mortality rate function and limiting the risk of overfitting. The continuous variable age was centred on 50.

#### Prediction of age- , sex- and deprivation-specific mortality

For both models, the deprivation- and age-specific death rates for each of the five deprivation categories were directly estimated from the sex-specific models for single years of age between 0 and 99. The death rates for England as a whole were derived from the weighted sum of each deprivation-specific mortality function, the weights being the proportion of each (age-deprivation) cell in the observed population.

#### Evaluation

For all four scenarios, the life tables were evaluated against the reference life tables. For the abridged data arrangements (i) and (ii), the Elandt-Johnson method was also evaluated (Fig. 1). The interpolation of the Elandt-Johnson method was applied on the proportions of survivors for each sub-population separately, i.e. by sex and deprivation category. The overall goodness-of-fit was assessed with the residual sum of squares (RSS). We also compared the value for the life expectancy at birth. Localised goodness-of-fit was also checked, using age-specific RSS and expectation of life (*e*_*x*_) at age 40 as well as for all ages from 0 to 100.

#### Findings

When using the smallest sample of data and the least detailed data arrangement, performance obtained with the Elandt-Johnson and flexible relational approaches was on average poorer than with the flexible Poisson approach, both overall and by age group (Table 2). Although the overall fit of the Elandt-Johnson and flexible relational approaches was not too divergent, estimates were consistently biased and the fit was mediocre for certain groups, in particular among the elderly (RSS over 5). This was also reflected in the substantial over-estimation of life expectancy, both at birth and at age 40, by almost 2 (Elandt-Johnson) and 5 years (flexible relational). By contrast, on average, life expectancy (at birth and at age 40) was over-estimated by less than one year when based on the flexible Poisson approach. This approach nevertheless did not always fit the data well and over-estimated the life expectancy at birth by up to 4.16 years. It was, however, much better than results from the two other approaches which over-estimated life expectancy at birth by over 11.65 years (Elandt-Johnson) and 13.54 years (flexible relational).

**Table 2:** Goodness-of-fit by approach: 1% sample and abridged data up to 80 years of age

		Elandt-Johnson	Flexible relational	Flexible Poisson
Residual Sum of Squares^a^				
All ages	min	0.001	0.003	2.8E-04
	mean	0.369	1.454	0.031
	max	5.866	8.914	0.607
Age 0–30	min	1.7E-05	1.3E-06	1.5E-05
	mean	0.001	0.011	0.001
	max	0.024	6.867	0.011
Age 31-79	min	4.2E-04	3.5E-04	1.7E-04
	mean	0.028	0.157	0.013
	max	0.322	0.860	0.158
Age 80-99	min	3.1E-05	0.001	2.0E-05
	mean	0.340	1.286	0.018
	max	5.781	8.836	0.585
Difference in life expectancy^b^ at:				
Birth	Min	0.001	0.040	0.001
	Mean	1.839	5.383	0.790
	Max	11.649	13.540	4.155
Age 40	Min	0.003	0.047	0.002
	Mean	1.804	5.313	0.662
	Max	11.829	13.379	3.843

^a^Mean, minimum and maximum difference in RSS across 100 simulated sets of deprivation-specific life tables compared to the reference tables

^b^Mean, minimum and maximum difference in life expectation (years) across 100 simulated sets of deprivation-specific life tables compared to the reference tables

When using the largest sample of the data and the most detailed data arrangement, the flexible Ewbank and the flexible Poisson approach provided fairly equivalent results, both in terms of overall fit and partial goodness of fit (mean and range of RSS, and life expectancy; Table 3). The flexible Poisson approach however displayed a slight advantage with smaller maximum RSS, overall and also amongst the elderly. By contrast, its performance was slightly less good for the younger ages. Both approaches tended to under-estimate the infant mortality rate, leading to an over-estimation of life expectancy at birth.

**Table 3:** Goodness-of-fit by approach: 20% sample and complete data up to 100 years of age

		Flexible relational	Flexible Poisson
Residual Sum of Squares^a^			
All ages	min	0.0E + 00	0.0E + 00
	mean	0.002	7.7E-05
	max	0.013	0.001
Age 0–30	min	0.0E + 00	0.0E + 00
	mean	5.1E-06	2.4E-05
	max	1.1E-04	2.2E-04
Age 31–79	min	0.0E + 00	0.0E + 00
	mean	0.002	4.2E-05
	max	0.012	0.001
Age 80–99	min	0.0E + 00	0.0E + 00
	mean	1.0E-04	1.1E-05
	max	0.001	3.9E-04
Difference in life expectancy^b^ at:			
Birth	Min	0.320	0.001
	Mean	0.498	0.069
	Max	0.679	0.209
Age 40	Min	0.259	2.0E-04
	Mean	0.478	0.038
	Max	0.650	0.154

^a^Mean, minimum and maximum difference in RSS across 100 simulated sets of deprivation-specific life tables compared to the reference tables

^b^Mean, minimum and maximum difference in life expectation (years) across 100 simulated sets of deprivation-specific life tables compared to the reference tables

**Fig. 2:**
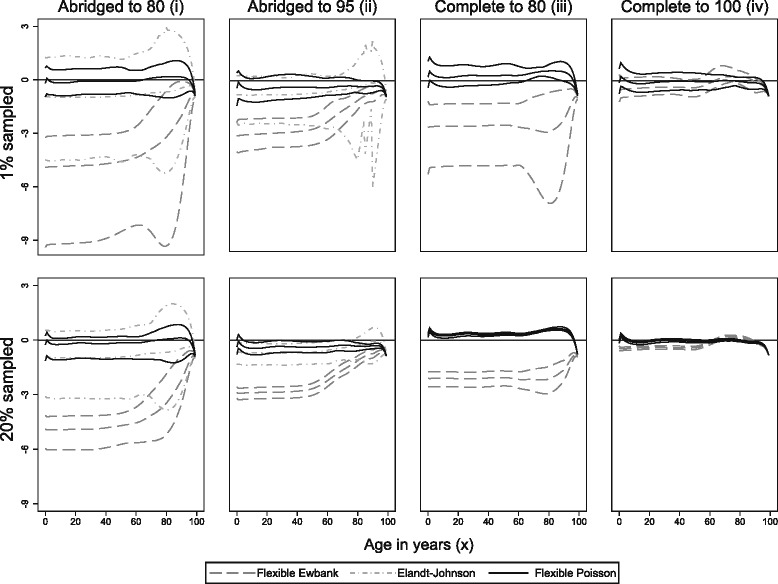
Differences in life expectancy estimates by sampled population and data arrangement. Mean, minimum and maximum difference in life expectation (years) across 100 simulated sets of deprivation-specific life tables compared to the reference tables

Figure 2 displays minimum, maximum and mean differences in mean age-specific life expectancy in the 100 national life tables. These are derived from the weighted average of the 100 sets of deprivation-specific tables. It shows that the performance of the flexible Poisson approach was consistently superior to the Elandt-Johnson approach (abridged data) and the flexible Ewbank approach (abridged and complete data), with the smallest and least biased errors in age-specific life expectancy.

All results for the 10% samples were very similar to the 20% samples. We also derived results from data containing a proportional effect of deprivation. The results from these analyses were similar to those derived from the non-proportional data.

### Evaluating an alternative approach for knot location

When fitting the flexible Poisson model in the simulation study we used *a priori* 6 knots for age and 3 for interactions. These were placed at the percentiles of the death distribution. We considered it likely, however that the fit of the model for the youngest ages might be improved based upon *a priori* knowledge about the pattern of mortality by age, which changes rapidly and very distinctly with age in the first 15 years of life: infant and child mortality is known to vary substantially between sub-populations.

#### Data

We obtained counts of deaths and populations in Wales (about 3,000 000 people) by single year of age, sex and calendar year for the period 1981–1991 from the Office for National Statistics.

#### Knot placement

Since the age-specific mortality is known to change most rapidly in the first days, weeks and years of life we fixed three knots *a priori* at ages 0, 1 and 2. It is known that inaccuracies in death and population counts arise amongst those over 85 even in countries with high quality demographic data. We thus grouped the data over 85 years of age and *a priori* located a knot at the median age of deaths in this age group. Further knots were also needed to catch the various inflexion points observed in the mortality rate between the ages of 2 and 85. We simulated 100 times random locations of 3, 4 and 5 additional knots between ages 3 and 50. This resulted in three series of 100 knot patterns (with 7, 8 or 9 internal knots, of which 4 were fixed *a priori*).

#### Modelling

We fitted models for each of the 300 patterns, separately for men and women, including only age in the model. The models were then ranked within each series according to their AIC. Among the models with the best fit (lowest AIC) and nearly equivalent (range of AICs lower than 3), it was possible to select a single pattern of knots which represented a common pattern amongst these models. Calendar year was added into the final model. We fitted an interaction between age and year *a priori*. We initially chose three internal knots for year and the interaction, located at the 25th, 50th and 75th centiles of their distributions. An algorithm based on AIC and embedded within the Stata command *mvrs* then selected the most appropriate number of knots for year and the interaction [11]. The variables were centred on age 50 and the central year in the data, 1986. The models were then used to predict complete, smoothed life tables for each sex and calendar year.

#### Findings

**Fig. 3:**
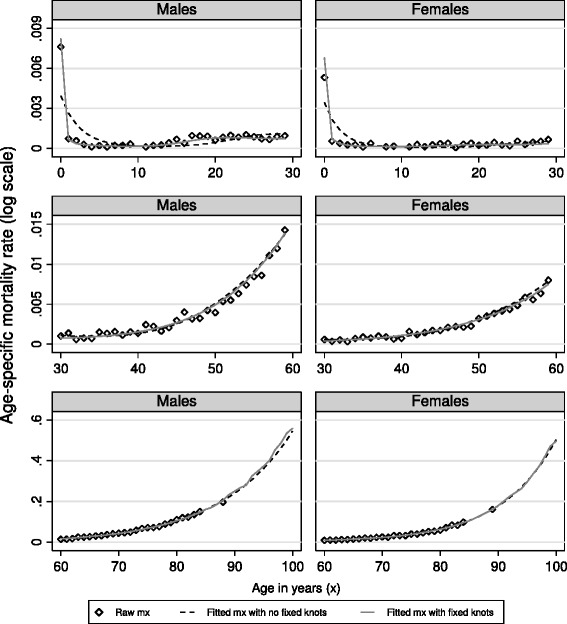
Age-specific mortality rates by age band and sex: raw values, fitted values obtained with no fixed knots, fitted values obtained with fixed knots. Knots were fixed *a priori* at ages 0, 1, 2 and at the median age at death over 85 years (see text)

We briefly describe here the results for men. The final model contained 8 knots for age (at 0, 1, 2, 14, 15, 27, 50, and 88 years) and three knots each for both year and the interaction. Figure 3 displays the fitted *m*_*x*_values for the raw data, the model with *a priori* fixed knots and the model with no fixed knots. The fit of the model with fixed knots was much better for the youngest ages but similar to the one with no fixed knots for ages over 30 years. The residual sum of squares for the survivorship curve (*l*_*x*_ function) comparing fixed and no fixed knots to the raw data were very small and very similar. Life expectancy at birth and at age 40 was also very similar to the raw data values. Differences ranged up to a maximum of 6 months.

## Discussion

We have shown that a flexible Poisson approach is equivalent or slightly better than the Elandt-Johnson approach for large samples, but that it is far superior to any other approach when estimating life tables for small populations. The superiority of this method is due to the fact that mortality rates are derived from actual counts of populations and deaths, and that the model is much more flexible. Further, we can assess the effect of several covariables and their potential interactions, rather than using a univariable approach. With the flexible Poisson approach outlying results are absent or exceptional, even where non-proportional effects are present. Where a proportional effect of deprivation was simulated the overall conclusion did not change (data available on request).

The Elandt-Johnson approach, currently recommended, uses a set of interpolations to derive fitted mortality rates. It displayed intermediate performance both overall and by age. It has been suggested that its performance was highest for national populations over the age range 20–99 years [8]. Our study shows however that the flexible Poisson model obtained much better results, for all ages from birth to 100 years.

Ewbank’s traditional relational model, and our flexible version of it, necessitates a standard population to derive the fitted rates. Despite the use of four parameters [3] instead of two [2], we have previously noticed a poor fit in young (ages 10–25) and old (ages 85+) ages. The flexibility gained with the addition of splines within a multivariable model improved the performance of the relational approach, but its results remain inferior to those obtained with the flexible Poisson model. In particular, the flexible relational model had a much higher proportion of outlying results. This is likely to be due to the fact that the approach uses the survivorship function rather than the actual counts of deaths and populations.

Both the Elandt-Johnson and the flexible Ewbank approach are based upon an underlying assumption about a universal shape of the human mortality profile by age. The use of a flexible Poisson model without fixed knots completely removes this assumption, because the knots for age are placed automatically according to centiles of the distribution of deaths. This resulted in a much better fit. Our subsequent evaluation of knot placement, in which certain knots were fixed whilst others were permitted to vary, resulted in an even better fit. Placing the knots in this manner introduces some *a priori* assumptions about the pattern of mortality by age, but these are minor compared with those imposed by the relational or interpolation approaches. In the present study, fixing some knots improved the accuracy of the estimates. Over-parameterisation is an issue with flexible functions such as splines and criteria other than AIC could have been used. However, our results hardly changed when using the Bayesian Information Criterion. Furthermore, in order to reduce the risk of over-parameterisation, we selected the final set of knots from among the models with best fit based on a recurrent pattern of knots. Further investigations could examine the placement of the knots at the oldest ages to better estimate mortality rates above the age of 85. The fact that *a priori* assumptions about the knot placement are not necessary but possible with the flexible Poisson model further strengthens the argument for its application over alternative approaches.

This study was conducted in England, which has a mortality profile representative of high-income countries. However, we have successfully used the approach to estimate life tables for 172 jurisdictions in 41 countries, spanning the five continents Africa, Central and South America, North America, Asia and Europe [19, 20]. There were a few situations where the fit was poor, in particular amongst the very young and the elderly. Other approaches were used for these populations but further improvement was rarely obtained.

Other flexible functions could be evaluated. Multilevel modelling could be investigated for scenarios when (one of) the co-variable(s) is a geographic area. These approaches can be assessed by a cross-validation process. Methodological developments have also been made within the actuarial framework [21–24], but these are fairly complex. It would be of interest to investigate their performance on sparse, poor-quality data as found in sub-populations and/or in low- and middle-income countries.

## Conclusions

Our recommended approach is that the flexible Poisson model should be used to model age-specific mortality rates of small populations, rather than conventional model life table techniques. Our approach is also more efficient in multivariable situations. The approach makes it possible to combine a prior understanding of the distribution of deaths by age (by the *a priori* location of certain knots for ages 0, 1, 2 and at the median age of death for those over 85) with a variable selection of knots (number and location) between the ages of 3 and 50, on the basis of simulations using the actual data.

Modelling the rates by sex is also preferable and simpler. When the data are in the abridged arrangement, the age variable should be centred for each group. In the case of poor quality data, the widely established Coale-Demeny standards [25] can be applied to estimate mean age of death for the population under 5 in abridged data.

Our results demonstrate the superiority of the flexible Poisson approach. We have shown that an approach which combines the flexibility of splines with a multivariable model can achieve a much better fit to the underlying mortality rates than approaches which make strong assumptions about the distribution of deaths by age.
